# Biomarkers for Diabetic Retinopathy – Could Endothelin 2 Be Part of the Answer?

**DOI:** 10.1371/journal.pone.0160442

**Published:** 2016-08-02

**Authors:** Nicolette Binz, Elizabeth P. Rakoczy, Ireni S. Ali Rahman, Nermina N. Vagaja, Chooi-May Lai

**Affiliations:** 1 Molecular Ophthalmology, Lions Eye Institute, Nedlands, WA, 6009, Australia; 2 Centre for Ophthalmology and Visual Science, The University of Western Australia, Crawley, WA, 6009, Australia; Justus-Liebig-University Giessen, GERMANY

## Abstract

**Purpose:**

The endothelins are a family of three highly conserved and homologous vasoactive peptides that are expressed across all organ systems. Endothelin (Edn) dysregulation has been implicated in a number of pathophysiologies, including diabetes and diabetes-related complications. Here we examined Edn2 and endothelin receptor B (Endrb) expression in retinae of diabetic mouse models and measured serum Edn2 to assess its biomarker potential.

**Materials and Methods:**

Edn2 and Ednrb mRNA and Edn2 protein expression were assessed in young (8wk) and mature (24wk) C57Bl/6 (wild type; wt), Kimba (model of retinal neovascularisation, RNV), Akita (Type 1 diabetes; T1D) and Akimba mice (T1D plus RNV) by qRT-PCR and immunohistochemistry. Edn2 protein concentration in serum was measured using ELISA.

**Results:**

Fold-changes in Edn2 and Ednrb mRNA were seen only in young Kimba (Edn2: 5.3; Ednrb: 6.0) and young Akimba (Edn2: 7.9, Ednrb: 8.8) and in mature Kimba (Edn2:9.2, Ednrb:11.2) and mature Akimba (Edn2:14.0, Ednrb:17.5) mice. Co-localisation of Edn2 with Müller-cell-specific glutamine synthetase demonstrated Müller cells and photoreceptors as the major cell types for Edn2 expression in all animal models. Edn2 serum concentrations in young Kimba, Akita and Akimba mice were not elevated compared to wt. However, in mature mice, Edn2 serum concentration was increased in Akimba (6.9pg/mg total serum protein) compared to wt, Kimba and Akita mice (3.9, 4.6, and 3.8pg/mg total serum protein, respectively; p<0.05).

**Conclusions:**

These results demonstrated that long-term hyperglycaemia in conjunction with VEGF-driven RNV increased Edn2 serum concentration suggesting Edn2 might be a candidate biomarker for vascular changes in diabetic retinopathy.

## Introduction

Diabetic retinopathy (DR) is a microvascular complication of the eye, where prolonged hyperglycaemia leads to capillary injury following changes to resident neuronal cell populations. Vascular endothelial growth factor (VEGF) becomes upregulated over time to replace lost and damaged capillaries in the retina. However, this process of neovascularisation produces dysfunctional neovessels and ultimately leads to tissue damage in the retina causing vision impairment and even loss. All diabetes sufferers are at risk of developing DR, which occurs in several stages from background and mild non-proliferative DR (NPDR) to proliferative DR (PDR), diabetic macular oedema and vision threatening DR, but only a subset are at risk of progressing to the severe forms of the disease. This subset of patients is currently estimated to be in the order of 66 million out of a projected 347 million diabetes patients worldwide [1, 2].

The gold standard of treatment for PDR has been laser photocoagulation (LPC) mainly to arrest and reverse neovascularisation [3, 4], despite the impact on peripheral and colour vision and the development or progression of macular oedema [5]. More recently, the use of anti-VEGF therapies has emerged as a promising novel treatment modality for PDR either alone or in conjunction with LPC [6]. However, these treatment options are reserved until PDR and/or macular oedema are established and tissue pathology evident on ocular examination. The challenge lies in finding biological markers that can identify those patients with NPDR at risk of retinopathy progression, ideally without the need for specialist ophthalmic examination, in a general practitioner setting at a low cost and most importantly, at a stage of the disease when little or no tissue pathology is present. The ability to identify patients at risk of progression using prognostic markers would benefit those patients through earlier intervention to preserve sight, and more directed treatment at a time point when it has the greatest impact on preventing disease progression prior to the development of significant tissue damage.

Current biomarkers such as duration of diabetes, glycated haemoglobin level (HbA1c), retinal examinations and albuminuria, for example, cannot detect early tissue damage [7]. Data from the 30 year DCCT/EDIC studies conducted by the National Institute of Diabetes and Digestive and Kidney Diseases (NIDDK) in the US [8] demonstrated the delaying effect of early intensive glucose control on the progression of DR with an impressive 50% reduction in the incidence of PDR in the intensive versus the conventional glucose control treatment group. However, 90–95% of patients in both treatment groups had some degree of retinopathy and 15% of patients in the intensive glucose control group still progressed to PDR during the course of the study. At present we lack definitive markers for retinopathy onset, development or progression and hence the ability to prevent DR and vision impairment. Biomarkers that would allow intervention at the subclinical stages of DR would be invaluable [9–11].

Methods for biomarker discovery vary widely and are continuously evolving. Novel biomarkers can be derived from genomic studies, investigations of disease-specific proteomes and metabolomes as well as from animal models of human diseases, for example. Here, we are evaluating a potential marker originally identified in a genome-wide microarray study on retinae from Kimba mice, a model of retinal neovascularisation (RNV) driven by photoreceptor-specific overexpression of VEGF [12–15] where we identified a novel association between VEGF upregulation and the vasoactive peptide endothelin 2 (Edn2) [16]. However, the Kimba mouse is normoglycaemic and hence we developed the hyperglycaemic Akimba mouse with neovascularisation by crossing the Kimba model of RNV with the Type 1 diabetic Ins2Akita mouse [17–20].

We examined Edn2 mRNA and protein expression across these three very distinct genotypes and phenotypes. The Kimba mouse is normoglycaemic, yet displays many of the retinal microvascular characteristics of human NPDR and PDR, including capillary drop-out, neovascularisation, microaneurysms, cotton wool spots and focal haemorrhages. The Akita mouse, with its spontaneous mutation in the insulin 2 gene, becomes hyperglycaemic at around 4 weeks of age, but does not develop any of the vascular changes associated with DR and RNV [17, 21]. The Akimba mouse is a combined genotype and phenotype of VEGF-induced RNV and hyperglycaemia. However, RNV in the Akimba mouse is more severe than in the Kimba mouse and exhibits the majority of signs of advanced clinical DR. While the Akimba mouse is not an ideal model for PDR it is very valuable in studying the combined effects of hyperglycaemia and neovascularisation [18, 19]. Duration of diabetes, i.e. hyperglycaemia, is a very important factor in the development of DR and hence we examined both young mice (8wk) after only four weeks of hyperglycaemia (short term) and mature mice (24wk), 20 weeks after the onset of hyperglycaemia (long term).

The aims of the current study were 1: to assess the effect of hyperglycaemia on Edn2 differential expression; 2: to determine whether in Akimba mice the combination of advanced RNV and hyperglycaemia would increase Edn2 expression above that observed in Kimba mice; 3: to establish the cellular localisation of Edn2 in the retinae of these animals deriving further clues to the retinal pathologies in these models; and 4: to assess whether mRNA changes in the retina translate into changes in circulating serum Edn2 protein, an important attribute of a potential biomarker.

## Materials and Methods

### Animals

Young (8wk) and mature (24wk) Akita (Ins2^Akita^), Kimba (trVEGF029) and Akimba (trVEGF029xIns2^Akita^) mice, all on the C57Bl/6 genetic background, were used throughout this study. Wt littermates were used as controls to ensure uniformity in genetic background, age and parental influence. Mice were housed at a constant temperature of 22°C with a 12:12 hour light/dark cycle [12, 13]. Food and water were available *ad libitum*. Genotyping was carried out as previously described [12]. Mice were euthanized by intraperitoneal injection of an overdose of sodium pentobarbital (Lethabarb; Virbac, NSW, Australia). All procedures were performed in accordance with the Association for Research in Vision and Ophthalmology (ARVO) statement for the ‘Use of Animals in Ophthalmic and Vision Research’ and with approval from the Animal Ethics Committee of The University of Western Australia, Australia.

### Blood Glucose Measurements and Glycated Haemoglobin Level

Mice were fasted as per Animal Models of Diabetic Complications Consortium guidelines and measurements were collected as previously described [18]. Blood glucose level (BGL) was determined using an Accu-Check Performa blood glucose meter (Roche Diagnostics Deutschland GmbH, Mannheim, Germany) with a range of 0.6 to 33.3mmol/L (10.8 to 599.4mg/dL). Readings above 33.3mmol/L were treated as 33.3mmol/L during analysis. HbA1c was determined using Siemen’s DCA2000+ Analyser (Siemens Medical Solutions Diagnostics, Bayswater Victoria, Australia) [18].

### Quantitative Real-time PCR

Total RNA was extracted from dissected retinae as previously described [16]. cDNA preparation, real-time PCR, preparation of standard curves, the use of peptidylprolyl isomerase A (Ppia) as the housekeeping gene and data analysis have been described previously [16].

### Immunohistochemistry

Five-micron thick paraffin sections were washed in phosphate-buffered saline and processed for staining with anti-Edn2, anti-glutamine synthetase (GS) and anti-glial fibrillary acidic protein (GFAP) antibodies as previously described [16]. All sections were counterstained with 4’-6’diamidino-2-phenylindole (DAPI; Roche) prior to confocal microscopy (Leica TCS SP II, Leica Microsystems GmbH, Wetzlar, Germany) at the Centre for Microscopy and Cellular Analysis, The University of Western Australia.

### Preparation of Serum Samples and Determination of Total Serum Protein Concentration

Blood was collected via intracardiac puncture into a 1ml syringe (Becton Dickinson Medical (S) Pte Ltd, Singapore) with a 25G 1” needle (Terumo Medical Corporation, Tokyo, Japan) and allowed to coagulate at room temperature (RT) in 2ml glass BD Vacutainer® tubes (BD, Franklin Lakes, USA). Samples were centrifuged for 15min at 1159rfc without braking, serum was transferred to a new tube for a second centrifugation at 1000rfc for 7min, both at RT, before being stored at -80°C. Total serum protein concentrations (mg/ml) were determined using the DC Protein Quantitation Assay (BioRad Laboratories Inc., Hercules, USA) on the Beckman Coulter AD200 Absorbance Detector (Beckman Coulter Inc., Fullerton, USA) to determine amount of Edn2 (pg) per mg of total serum protein.

### Edn2 ELISA

Six mice per group (wt, Akita, Kimba and Akimba at 8 and 24wks-of-age) were analysed using the Endothelin-2 ELISA Kit (Phoenix Pharmaceuticals Inc., Burlingame, USA) as previously described [16].

### Data Analyses

Following single factor ANOVA at α = 0.05, multiple comparisons were made using Tukey’s multiple comparison test. Effect of age and genotype on Edn2 serum concentrations was determined using two-factor ANOVA at α = 0.05. All data analyses were performed using Prism 5 (Version 5.0c; GraphPad Software Inc., La Jolla, USA). Data are presented as mean ± SEM.

## Results

### Metabolic Parameters: Normoglycaemic wt and Kimba mice differed significantly from hyperglycaemic Akita and Akimba mice

There was a clear demarcation across all physiological and metabolic parameters between normoglycaemic (wt and Kimba) and hyperglycaemic (Akita and Akimba) young (8wk) and mature (24wk) mice (Table 1). In Akita and Akimba mice there was no significant weight gain over time compared to age-matched wt and Kimba mice. Even short term-hyperglycaemia of only 4 weeks duration lead to measurably lower body weights and significantly greater kidney to body weight ratios (p<0.05) in young Akita (7.7 ±0.3) and Akimba mice (7.1 ±0.2) compared to young wt (6.3 ±0.1) and Kimba mice (5.9 ±0.1). The ratios reported here for wt and Kimba mice are in line with those presented by Gurley et al. [22] of 5.95 ± 0.11mg/g for C57Bl/6 mice, the genetic background of Akita, Kimba and Akimba mice. Compared to the wt and Kimba mice there was also an increase in kidney to body weight ratios in Akita (p<0.05) and Akimba (p<0.005) mice with age and increasing duration of hyperglycaemia (Table 1). BGL was significantly elevated in young Akita (28.5±1.6mmol/L; p<0.005) and Akimba mice (26.8±1.2mmol/L; p<0.005) after only 4 weeks of hyperglycaemia compared to wt (13.1±0.6mmol/L; p<0.005) and Kimba mice (13.4±0.5mmol/L; p<0.005). While BGL in wt and Kimba mice was elevated compared to previously published data for C57Bl/6 mice [22], corresponding HbA1c levels were normal, indicating that these animals were normoglycaemic and there was no increase in BGL or HbA1c over the study period. HbA1c was already significantly elevated (p<0.005) in young Akita (7.5±0.3%) and Akimba (7.1±0.3%) mice compared to wt (4.3±0.1%) and Kimba mice (4.4±0.1%). HbA1c waswas elevated further with increasing age, which also meant duration of hyperglycaemia, in Akita (11.2±0.4%) and Akimba mice (10.4±0.4%), while wt and Kimba HbA1c remained the same with increasing age. This suggested that hyperglycaemia, not RNV, was responsible for the changes such as increase in HbA1c, decrease in body weight and increase in kidney to body weight ration observed in Akita and Akimba mice (Table 1).

**Table 1:** Physiologic and metabolic parameters of young and mature wt, Kimba, Akita and Akimba mice.

Genotype	Age wks ^1^	BGL (mmol/L)	BGL (mg/dL)	HbA1c (%)	HbA1c (mmol/mol)	Body Weight (g)	Kidney to Body Weight ratio
							(mg/g)
Wt	Y(27)	13.1^2^ (0.6)	237 (11.1)	4.3 (0.1)	24 (1)	21.4 (0.4)	6.3 (0.1)
	M(25)	13.52 (0.7)	243 (12.4)	4.8 (0.1)	29 (1)	28.7 (0.3)	5.9 (0.1)
K	Y(23)	13.42 (0.5)	241 (9.2)	4.4 (0.1)	24 (1)	21.3 (0.4)	5.9 (0.1)
	M(10)	8.8 (0.8)	159 (15.0)	4.4 (0.1)	25 (1)	27.1 (0.6)	6.2 (0.3)
A***	Y(10)	28.5^3^ (1.6)	514 (25.4)	7.5 (0.3)	59 (4)	19.0 (0.4)	7.7 (0.3)
	M(26)	28.1^3^ (1.0)	507 (17.3)	11.2 (0.4)	99 (4)	20.2 (0.4)	9.1 (0.2)
AK***	Y(13)	26.8^3^ (1.2)	483 (21.9)	7.1 (0.3)	54 (3)	18.5 (0.4)	7.1 (0.2)
	M(34)	28.1^3^(1.0)	507 (17.9)	10.4 (0.4)	91 (5)	20.6 (0.3)	8.7 (0.2)

^1^ Numbers in brackets represent numbers of animals per group; Y = young, M = mature

^2^ While BGL in wt and Kimba mice was elevated compared to previously published data for C57Bl/6 mice (20), HbA1c levels were normal indicating that these animals were normoglycaemic.

^3^ The Blood glucose meter was not able to read above 33.3mmol/L. In Akita (8 and 24wks) and Akimba (8 and 24wks) mice, between 10–36% had readings above the meter’s limit, hence actual average BGL will be higher than reported here. HbA1c is therefore a more accurate representation of BGL over the preceding two months.

Duration of hyperglycaemia: in Akita and Akimba mice, onset of hyperglycaemia occurred at around 4 weeks of age; in young mice at 8weeks of age, the mice have only experienced short-term hyperglycaemia, while mature mice have been exposed to long-term hyperglycaemia (20 weeks).

***p<0.005 in A and AK mice compared to wt and K mice for all metabolic and physiological parameters examined. All data are represented as mean±SEM.

### mRNA expression of Edn2 and its receptor Ednrb was significantly upregulated in the presence of RNV and hyperglycaemia but not hyperglycaemia alone

**Fig 1:**
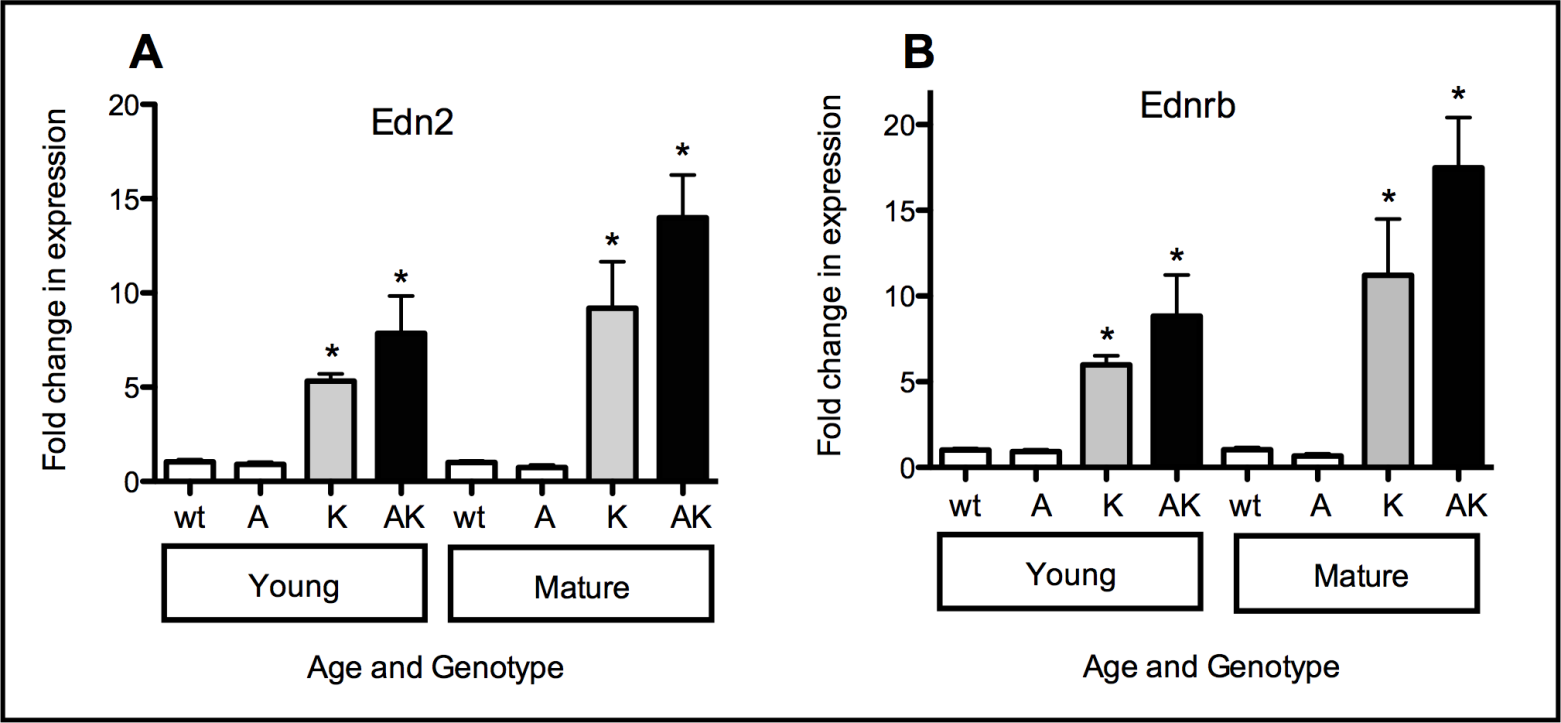
**Edn2 (A) and Ednrb (B) fold changes in mRNA expression in young and mature retinae of wt, Akita, Kimba and Akimba mice**. Data represented are fold changes in expression normalised against Ppia as the housekeeping gene. Differential expression was determined using delta delta Ct according to Livak and Schmittgen [23]. Fold changes in expression in Akita, Kimba and Akimba retinae represent increases or decreases in mRNA expression levels compared to wt retinae. N = 4 per group; *p<0.05 compared to wt and Akita

We assessed the effect of hyperglycaemia on mRNA expression of Edn2 and Ednrb. Hyperglycaemia alone did not affect Edn2 and Ednrb mRNA expression in retinae of Akita mice neither in young mice after only short-term hyperglycaemia nor in mature mice after long-term hyperglycaemia compared to wt littermates. The Edn2 mRNA fold-changes in young and mature Akita mice were 0.9 and 0.7, respectively (Fig 1A). The corresponding Ednrb mRNA fold-changes in young and mature Akita were: 0.9 and 0.7, respectively (Fig 1B; p>0.05). The presence of the VEGF_165_ transgene alone (Kimba) and the combination of transgene and hyperglycaemia in Akimba mice led to significant fold-change increases in Edn2 mRNA (Kimba: 5.3; Akimba: 7.9, p<0.05, Fig 1A) and Ednrb (Kimba: 6.0; Akimba: 8.8, p<0.01, Fig 1B) expression even in young mice, although the difference between young Kimba and Akimba mice was not significant. mRNA expression for Edn2 and Ednrb continued to increase in both Kimba and Akimba mice with age, but these were also not statistically significant (p>0.05; Fig 1A and Fig 1B).

### Photoreceptors and Müller cells are the primary expression sites for Edn2 in the retina

**Fig 2:**
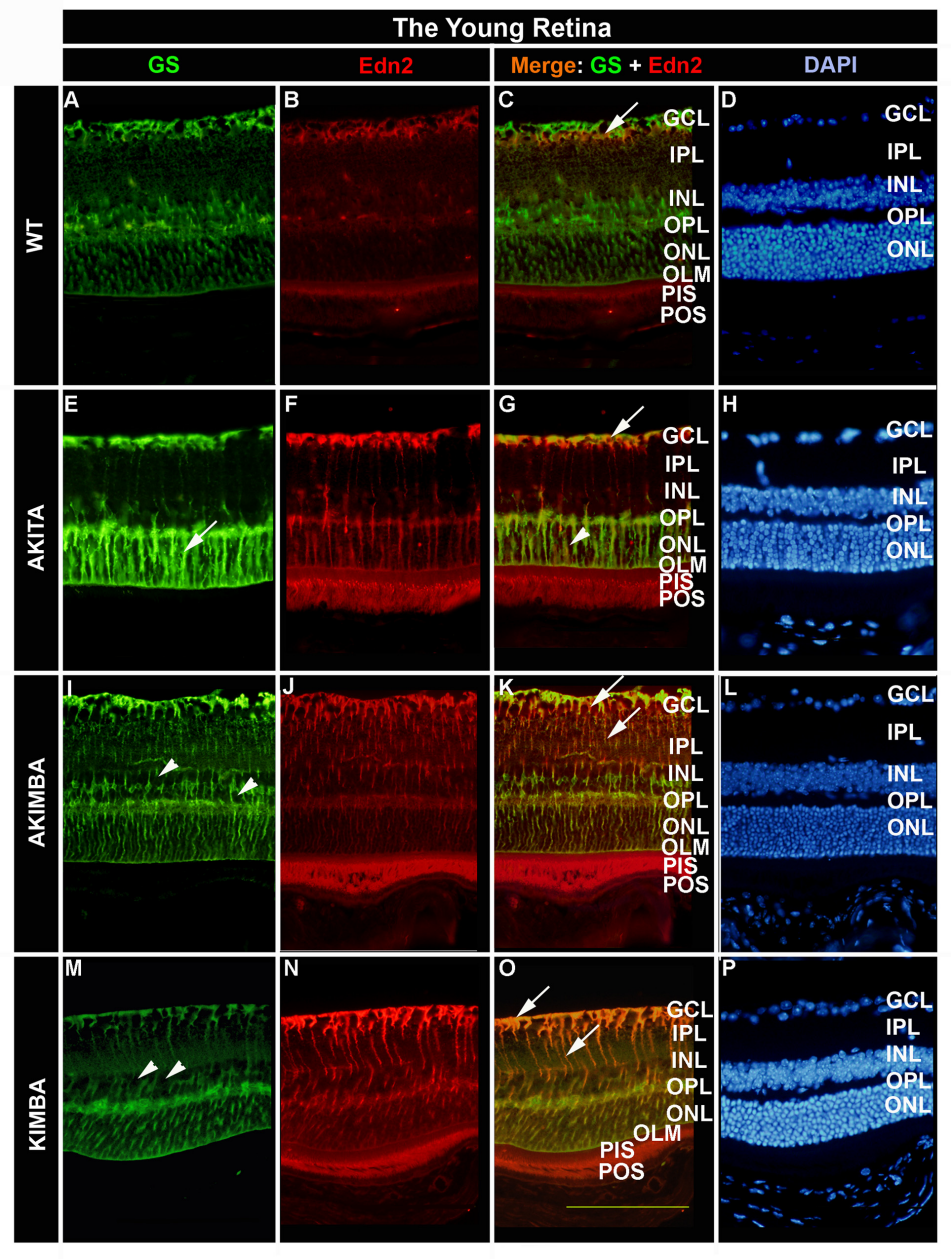
Cellular localisation of Edn2 in the young retina. Edn2 expression was localised to Müller cells as demonstrated by co-localisation with glutamine synthetase (GS), a Müller cell-specific enzyme, and photoreceptor inner and outer segments (PIS, POS) in wt, Akita, Akimba and Kimba retinae (C, G, K, O; arrows and arrowhead). In Akita, Edn2 expression was mostly in the GCL, where Müller cell endfeet reside, the outer plexiform layer (OPL) and the outer nuclear layer (ONL; E, G). In Akimba and Kimba, Edn2 expression was mostly in the inner retina, the GCL and the inner plexiform layer as well as the photoreceptor inner and outer segments (IPL, PIS, POS; J, K, N, O). Co-localisation of Edn2 and GS was most pronounced in the GCL and Müller cell processes in the IPL of Kimba mice (O). Scale bar: 100μm

In young wt retinae, Edn2 protein was expressed in the ganglion cell layer (GCL), the outer plexiform layer (OPL) and the photoreceptor inner segments (PIS; Fig 2B). Co-staining with glutamine synthetase (GS), a Müller cell-specific enzyme, showed co-localisation of Edn2 and GS protein expression restricted to the GCL (Fig 2C arrow) in wt mice, with added co-staining in the outer nuclear layer (ONL) in Akita retinae (Fig 2G arrow and arrowhead). GS protein expression in Müller cells was least in the inner plexiform layer in young wt mice (IPL; Fig 2A). The pattern for GS protein staining was similar in young Akita mice with strong immunoreactivity in the GCL, OPL and particularly the ONL (Fig 2E arrow), suggesting that even short-term hyperglycaemia activated Müller cell neuroprotective behaviour in support of photoreceptors in the hyperglycaemic Akita retina. Edn2 protein expression was prominent in the GCL, OPL and ONL as well as the PIS and photoreceptor outer segments (POS) in these mice, with strong co-expression in the Müller cell endfeet found in the GCL, abutting the inner limiting membrane of the retina (Fig 2G, arrow). As in young wt mice, Edn2 protein staining was least in the IPL and inner nuclear layer (INL; Fig 2F). The major difference between young wt and Akita retinae compared to age-matched Akimba and Kimba mice was the prominent GS protein expression in all parts of resident Müller cells, from the inner limiting membrane to the outer limiting membrane (OLM) of the retina, including the IPL and INL (Fig 2I and Fig 2M) in the presence of RNV. The same was true for Edn2 protein staining in young Akimba and Kimba retinae (Fig 2J and Fig 2N) with strong co-expression of Edn2 and GS in Müller cells most prominently in the inner retina, the GCL and IPL, and the Müller cell processes in these layers (Fig 2K and Fig 2O, arrows).

**Fig 3:**
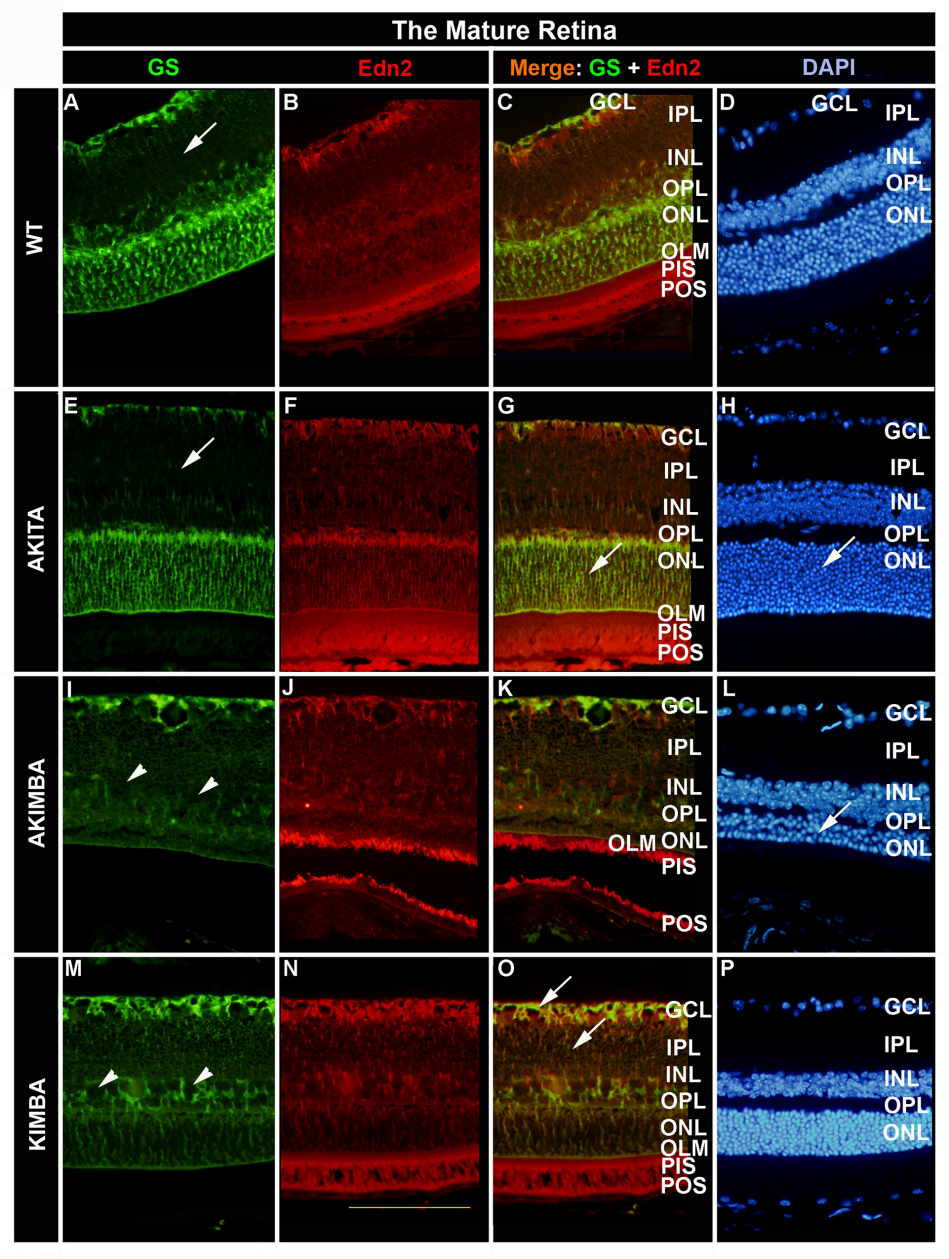
Cellular localisation of Edn2 in the mature retina. Following 20 weeks of chronic hyperglycaemia in the mature Akita retina there was increased Edn2 staining in the IPL compared to young Akita retinae (F). Particularly in the mature Akimba retina GS staining was much reduced compared to the young retina, suggesting not only loss of photoreceptors (L) but also loss of Müller cells (I, arrowheads). Müller cell loss was also evident in mature Kimba retinae (M, arrowheads; O, arrows) compared to young Kimba retinae, but the loss was less pronounced. Scale bar: 100μm

The expression pattern for both Edn2 and GS proteins did not change in mature wt and Akita mice compared to young mice, with the IPL and INL portions of Müller cells showing very little or no GS immunoreactivity (Fig 3A and Fig 3E; arrows). Long-term hyperglycaemia had no worsening effect on Müller cell integrity in Akita mice, though the extensive GS protein staining throughout the ONL was striking (Fig 3G, arrow). In contrast, mature Akimba retinae displayed a loss of GS activity throughout the retina, raising the question of Müller cell loss in these mice. The strong immunoreactivity seen in Akita ONL was completely missing in Akimba retinae of the same age (Fig 3I arrowheads) following the same duration of hyperglycaemia. The ONL may be much reduced in mature Akimba compared to Akita retinae, but photoreceptors are still present (Fig 3H and Fig 3L arrows). Mature Kimba retinae retained much of the staining pattern of young retinae, although Edn2 protein staining in the GCL and IPL appeared reduced with less GS co-staining in the innermost layers of the retina (Fig 3O arrows).

The presence of RNV in Kimba and Akimba retinae did produce a different expression pattern for both Edn2 and GS compared to hyperglycaemic Akita retinae. In the diabetic Akita retina, Edn2 staining was most pronounced in Müller cell endfeet in the inner retina, Müller cell processes in the OPL and throughout the ONL, as well as the inner and outer segments of the photoreceptors (Fig 2F), a pattern that was maintained even after long-term hyperglycaemia in mature diabetic retinae (Fig 3F). In contrast, young Akimba mice exhibited more Edn2 expression in Müller cell processes found in the inner retina, the IPL and INL in addition to the photoreceptor inner and outer segments (Fig 2J). Interestingly, staining in the ONL was less marked in the Akimba retina compared to the Akita retina.

**Fig 4:**
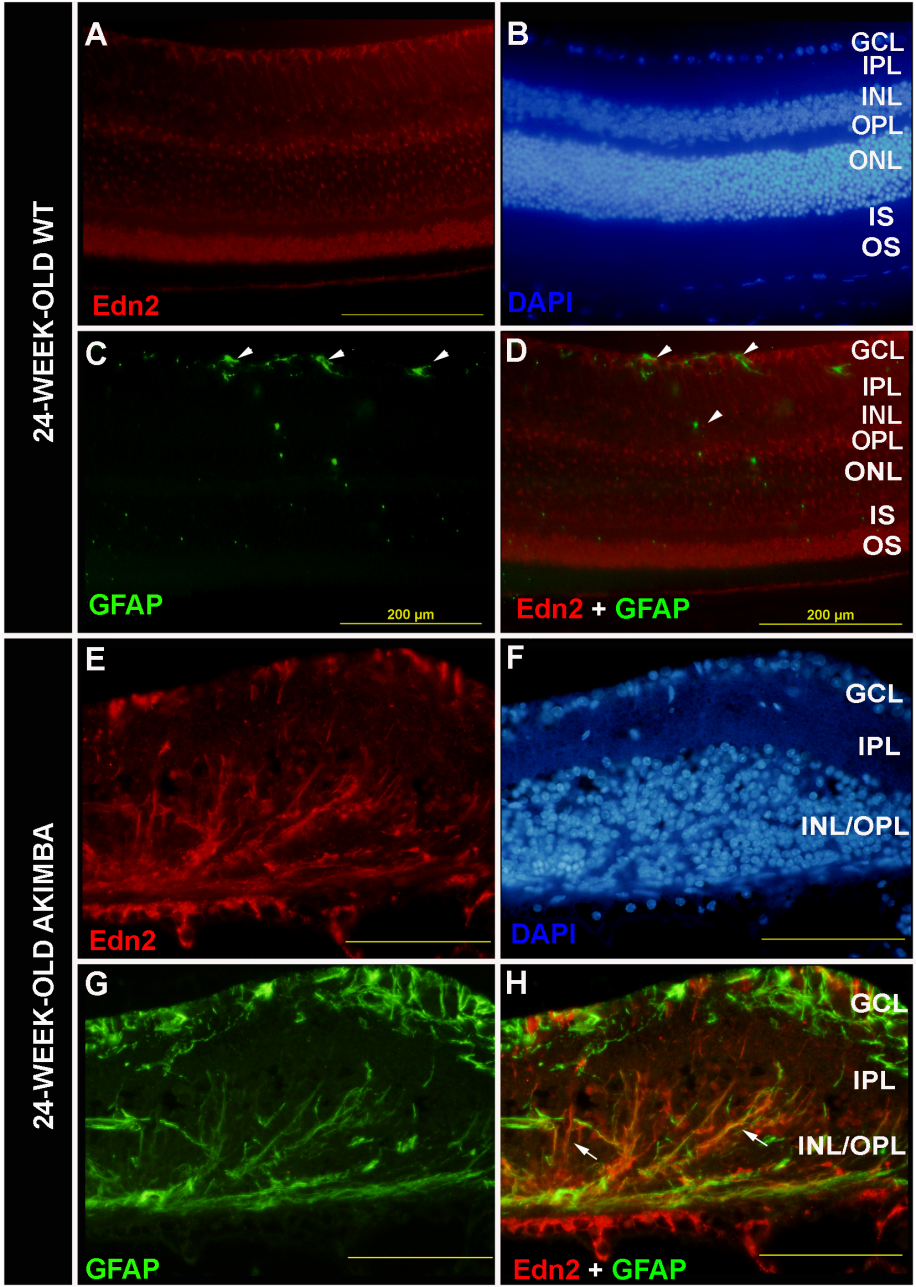
Evidence of Müller cell gliosis in mature Akimba mice with a severe phenotype. In the mature wt retina GFAP expression was localised and restricted to the astrocytes that reside on the GCL (arrowheads, C-D). In Akimba mice, GFAP expression was observed as thick processes in the GCL and long radial processes in the INL/OPL, indicating Müller cell gliosis (G-H). Edn2 only co-localised with GFAP in Müller cell processes in the INL/OPL (arrows, H) but not in the astrocytes of the GCL. Sections were counterstained with DAPI (B, F). GCL: ganglion cell layer; IPL: inner plexiform layer; INL: inner nuclear layer; OPL: outer plexiform layer; ONL: outer nuclear layer; PIS/POS: photoreceptor inner and outer segments. Scale bar: 200μm

We also examined Müller cell gliosis in mature Akimba mice with severe retinal pathology in comparison to mature wt retinae. As expected, GFAP protein staining in mature wt retinae remained localised to astrocytes resident on the nerve fibre layer of the GCL (Fig 4C and Fig 4D; arrowheads) with no staining in Müller cell processes indicating Müller cell quiescence under normal physiological conditions. In contrast, in the mature Akimba retina with severe retinal pathology (Fig 4E—Fig 4H) GFAP protein expression was observed as thick, reactive processes in the GCL and long radial processes in the INL/OPL, demonstrating Müller cell gliosis.

### Edn2 protein concentration in sera from mature Akimba mice was significantly increased compared to mature wt, Kimba and Akita mice

**Fig 5:**
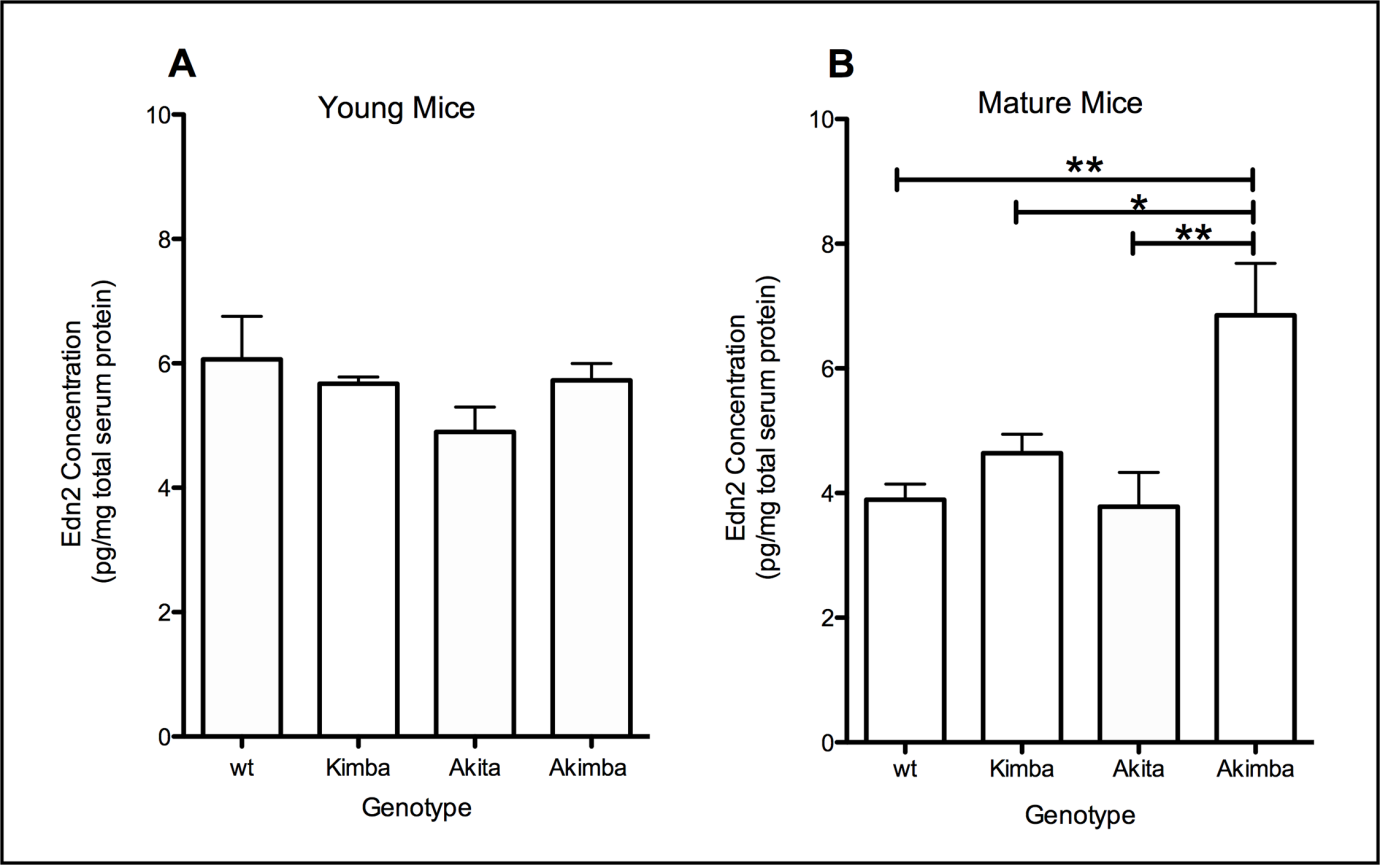
Serum Edn2 concentration in young and mature wt, Akita, Kimba and Akimba mice. Serum concentrations did not differ between young wt, Akita, Kimba and Akimba mice (A). In wt mice, Edn2 serum concentration decreased with age. In mature mice (B), there was no significant difference in serum Edn2 concentration between wt, Akita and Kimba mice. However, circulating levels of Edn2 were significantly higher in mature Akimba mice (6.9pg/mg total retina protein) compared to wt (3.9pg/mg; p<0.01), Akita (3.8pg/mg; p<0.01) and Kimba (4.6pg/mg total retina protein; p<0.05) mice.

Serum Edn2 protein concentrations decreased significantly with age in wt mice (p<0.05), but not in Kimba, Akita or Akimba mice (Fig 5A and Fig 5B). In mature mice, serum Edn2 protein concentration was significantly increased in Akimba compared to age-matched wt (p<0.001), Kimba (p<0.05) and Akita (p<0.001) mice. Thus, RNV combined with diabetes had the most significant effect on circulating Edn2 protein concentrations. Two-way ANOVA showed that genotype, not age, was the determining factor affecting serum Edn2 concentration in this study (p<0.0001).

## Discussion

The endothelins are a family of three highly conserved and homologous vasoactive peptides that are expressed across all organ systems and their dysregulation has been implicated in a number of pathophysiologies [6, 24–31], including diabetes and diabetic complications [32–38]. Despite being encoded by separate genes on different chromosomes, these three peptides (ET-1, ET-2 and ET-3) show very high sequence homology, particularly ET-1 and ET-2 [39]. At present it is assumed that ET-1 and ET-2 also bind to the two main endothelin receptors, ET_A_ and ET_B_, with similar affinity, and hence little distinction has been made between the two isoforms [40]. However, Ling et al. [31] recently commented on the converging evidence for an important and distinct ET-2 pathway, calling for a re-evaluation of the endothelin family. This hypothesis is supported by research using transgenic mice where ET-2 knockout mice displayed a phenotype distinct from that of ET-1 or ET-3 knockout mice [40]. Furthermore, there have been a host of studies examining gene expression changes in the retina in response to hyperoxia, retinal degeneration, photoreceptor cell loss and retinal detachment [41–45]. In each instance, Edn2 was one of the most significantly upregulated genes and the only isoform to be differentially regulated, mostly early in the disease process, and hence Edn2 has been described as an early stress response gene. This is in line with our own observations of Edn2 mRNA upregulation in retinae of 10d old Kimba mice [16].

In the present study we confirmed that Edn2 and Ednrb mRNAs were upregulated in both Kimba and Akimba mice. We have also shown that this upregulation persisted in mature mice, when vasculopathy was well advanced in Akimba mice. Interestingly, Ednrb mRNA expression changed in accordance with that of its ligand, Edn2. Thus, there is the potential for increased Edn2 affecting downstream metabolic processes, particularly in light of our previous demonstration of increased Edn2 protein in whole eye extracts [16]. There was no increase in Edn2 and Ednrb mRNA expression in young or mature Akita mice, suggesting that VEGF, not hyperglycaemia, was the driving factor in the upregulation of Edn2 activity in retinae of these mice. This is supported by the lack of vascular changes reported in retinae of young and mature Akita mice [17, 18]. The presence of mild neuronal changes in mature Akita mice was not sufficient to induce Edn2 or Ednrb mRNA production.

Photoreceptors and Müller cells were the primary expression sites for Edn2 in retinae of young and mature Kimba, Akita and Akimba mice. The retinal architecture in the mature Akimba retina was markedly different to that of the mature Akita and young Akimba retina. Significant photoreceptor cell loss coupled (Fig 3L) with reduced GS reactivity in these retinae (Fig 3I) suggested loss of Müller cell integrity if not loss of Müller cells themselves (Fig 3I arrowheads). The cell loss appeared greater in Akimba than in mature Kimba retinae (Fig 3M–Fig 3P, arrowheads). These images are similar to those presented on a model of Müller cell ablation by Shen et al. [46], showing characteristic ‘holes’ in the INL presumably where Müller cell bodies were resident prior to ablation. Diabetes and RNV produced different staining patterns for Edn2 expression in the retina, yet both led to Müller cell reactivity and support the concept of photoreceptor cell stress being induced in Akita, Kimba and Akimba mice. The important difference was that hyperglycaemia in the absence of vascular disease did not lead to upregulation of Edn2, but excess VEGF and the resultant RNV did.

Müller cells have been described as the core of a columnar ‘micro-unit’ of retinal neurons essentially providing structural as well as metabolic support to the photoreceptors to which they are anatomically connected [47]. Müller cell gliosis is evident in virtually every retinal pathology, however, it is still unclear what happens first: are Müller cells activated and sometimes lost in response to photoreceptor cell stress and subsequent death, or alternatively, are Müller cells lost early on, leaving the photoreceptors within their ‘micro-unit’ without neurotrophic support and neuroprotection, leading to photoreceptor cell loss? In diabetic retinopathy early neuropathology is followed by the development of RNV. In Akita mice early neuronal changes have been described [17], although they do not include photoreceptor cell loss [21]. However, increased glial fibrillary acid protein (GFAP) expression in Müller cells has been reported [21], suggesting that in the Akita mouse earliest cellular changes in response to hyperglycaemic stress consist of Müller cell activation. In the Akimba mouse, hVEGF_165_ transgene expression commences upon photoreceptor maturation by postnatal day 7 and we know from studies on 10d old Kimba mice that even three days of transgene expression were sufficient to induce pathological changes in retinae of these mice, including Edn2 and Ednrb upregulation [48]. Hyperglycaemia, on the other hand, does not commence until postnatal week 4 in Akita and Akimba mice, hence in Akimba mice excess VEGF causing photoreceptor stress and RNV would precede neuropathology in response to hyperglycaemia. Rattner and Nathans suggested that photoreceptor injury invariably leads to Müller cell activation, which is the case in Akimba mice, and that the co-expression of Edn2 in both Müller cells and photoreceptors together with the Edn2 receptor Ednrb points to a molecular system for communication between Müller cells and photoreceptors, allowing Müller cells to monitor retinal neurons for signs of injury or stress [48, 49]. This retinal injury monitoring system is activated early in both Kimba and Akimba mice, but not in Akita mice.

Studies on young normoglycaemic Kimba mice have shown that RNV also leads to increased GFAP expression and reactive Müller cell gliosis, including scar formation along the OLM [48]. Based on the present data we suggest that Müller cell loss occurs in addition to photoreceptor cell loss in retinae of these mice, and that this cell loss is most marked in mature Akimba mice (Fig 4G). Photoreceptor cell loss was evident in young Akimba retinae while there was still strong immunoreactivity for GS in Müller cells and retinal architecture was still well organised (Fig 2I–Fig 2L), supporting the hypothesis that Müller cells were lost following photoreceptor cell death, resulting in a cellularly disorganised retina with considerable GFAP reactive Müller cell gliosis in the mature Akimba retina (Fig 4G and Fig 4H). In the Akimba retina we observed the early activation of the Edn2-Ednrb injury-related signalling pathway between photoreceptors and Müller cells, with increased expression at the mRNA level maintained to 24 weeks of age in mature mice, despite significant glial and neuronal cell loss. However, this response was not sufficient to prevent the severe retinal injury caused by the combination of early-onset RNV and chronic hyperglycaemia in mature Akimba mice. At present we do not know whether the increase in circulating Edn2 protein in mature Akimba mice is solely the result of a more severe retinal phenotype, whether it is due to the combination of RNV and long-term hyperglycaemia, or whether in fact DN also contributed to the elevation of serum Edn2 protein Future studies on animal models will be required to clarify these questions and to determine whether Edn2 might be part an early warning system to identify patients at risk of progressing from background DR to PDR.

## Conclusions

These results demonstrated that long-term hyperglycaemia in conjunction with VEGF-driven RNV increased Edn2 serum concentration suggesting Edn2 might be a candidate biomarker for vascular changes in diabetic retinopathy.
